# An Efficient Blue‐Emission Crystalline Thin‐Film OLED Sensitized by “Hot Exciton” Fluorescent Dopant

**DOI:** 10.1002/advs.202203997

**Published:** 2022-11-17

**Authors:** Jingjie Yang, Wantao Zheng, Dehua Hu, Feng Zhu, Yuguang Ma, Donghang Yan

**Affiliations:** ^1^ State Key Laboratory of Polymer Physics and Chemistry Changchun Institute of Applied Chemistry Chinese Academy of Sciences Changchun 130022 China; ^2^ School of Applied Chemistry and Engineering University of Science and Technology of China Hefei 230026 China; ^3^ State Key Laboratory of Luminescent Materials and Devices South China University of Technology Guangzhou 510640 China

**Keywords:** crystalline molecular thin films, doping, hot exciton material, OLED

## Abstract

Crystalline thin‐film organic light‐emitting diodes (C‐OLEDs) can achieve a large light emission and a low Joule‐heat loss under low driving voltage due to the high carrier mobility of the crystalline thin films. However, it is urgent for the C‐OLEDs to improve their external quantum efficiency (EQE). Here, a novel strategy is proposed using a doped “hot exciton” material to sensitize a high PLQY blue emitter in C‐OLEDs. Benefiting from the capability of the “hot exciton” material harnessing triplet/singlet excitons, the C‐OLED exhibits an efficiency breakthrough with a maximum EQE of 6.2%, a much enhanced blue photon output with pure blue emission Commission International de L'Eclairage (CIE) (0.14, 0.15), a low turn‐on/operation voltage of 2.6 V(@1 cd m^−2^)/3.8 V (@1000 cd m^−2^), and a maximum power efficiency (PE) of 9.4 lm W^−1^. This work unlocks the potential of C‐OLEDs for achieving high photon output with high EQE.

## Graphical abstract

By employing crystalline organic semiconductor as host matrix and doped “hot exciton” material as sensitizer, an efficient crystalline thin‐film blue‐emission organic light‐emitting diode with high photon output and low driving voltage is created.

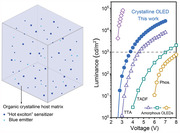

## 1 Introduction

In the past decades, organic light‐emitting diodes (OLEDs) have aroused great interest in full‐color displays and white lighting for their potential advantages of flexibility, fast response, low power consumption, and flat emission.^[^ ^1^, ^2^ ^]^ Although amorphous thin‐film forms are widely used in current high‐efficiency OLEDs, the disordered molecular arrangement in the amorphous materials leads to low carrier mobility, restricting the carrier transport capability and photon output power. Therefore, there is an essential requirement, but a critical challenge, to realize high photon output with sufficient efficiency under low driving voltage. Since electroluminescence was first observed in anthracene single crystals in 1963,^[^ ^3^ ^]^ continuous efforts have been spent on light‐emitting devices made of organic crystalline materials,^[^ ^4^, ^5^, ^6^, ^7^, ^8^, ^9^ ^]^ such as single‐crystal OLEDs,^[^ ^10^ ^]^ single‐crystal organic light‐emitting transistors (OLETs),^[^ ^11^, ^12^ ^]^ and crystalline thin‐film OLEDs (C‐OLEDs) fabricated with weak‐epitaxy‐growth technology.^[^ ^8^, ^13^, ^14^, ^15^ ^]^ C‐OLEDs with fluorescent materials have shown promising blue emission because of the host's intrinsic properties of high carrier mobility.^[^ ^15^ ^]^ However, due to the spin‐statistics only singlet excitons can decay radiatively,^[^ ^16^, ^17^ ^]^ and the external quantum efficiency (EQE) of these reported C‐OLEDs was less than 5%, although high photoluminescence quantum yields (PLQY) blue dopant has been introduced.^[^ ^15^ ^]^ To further improve the EQE and photo output capability of C‐OLEDs, it is worth considering a strategy of utilizing sensitizers to harvest and transfer more singlet/triplet excitons to the doped blue fluorescent emitters.^[^ ^18^, ^19^, ^20^, ^21^, ^22^, ^23^, ^24^ ^]^ “Hot exciton” materials are able to achieve almost 100% singlet exciton yield by the reverse intersystem crossing process from high‐lying triplet states (hRISC) to singlet excited states.^[^ ^25^, ^26^, ^27^, ^28^, ^29^, ^30^, ^31^, ^32^, ^33^ ^]^ The fast hRISC process will effectively reduce the quenching process related to long‐lived triplet excitons (e.g. T_2_ − T_1_). Different from the often quenching of the long‐lifetime triplet excitons in thermally activated delayed fluorescence (TADF)^[^ ^34^ ^]/^phosphorescence materials^[^ ^35^ ^]^ and the theoretically maximum IQE of 62.5% in triplet‐triplet annihilation (TTA) materials,^[^ ^36^, ^37^ ^]^ “hot exciton” materials can both achieve a high exciton utilization efficiency and a low‐efficiency roll‐off of OLEDs. Thus, the “hot exciton” channel sensitizing fluorescent emitter is expected to achieve a high yield of exciton utilization for ideal blue fluorescent emissions in C‐OLEDs.

In this work, we report a new type of double‐doped system C‐OLED, composed of a crystalline wide‐bandgap fluorescent host matrix, a doped “hot exciton” material as a sensitizer, and a doped high‐PLQY blue fluorescent material as an emitter. The device, benefiting from the high carrier mobility of crystalline thin film and the effective exciton collection capability of “hot exciton” material, exhibits an efficient blue fluorescent emission (Commission International de L'Eclairage (CIE) (0.14, 0.15)) with a low turn‐on/operation voltage of 2.6 V(@1 cd m^−2^)/3.8 V(@1000 cd m^−2^) and a maximum EQE up to 6.2%. In contrast with representative reported amorphous thin‐film blue OLEDs (CIE_y_ ≤ 0.15) with high EQE values, the double‐doped C‐OLED shows a lower ratio of series resistance Joule heat to input power, and a dominant capability of blue photon emission at limited operation voltage, proving the superiority of the crystalline route in OLEDs.

## 2 Results and Discussion

**Figure 1:**
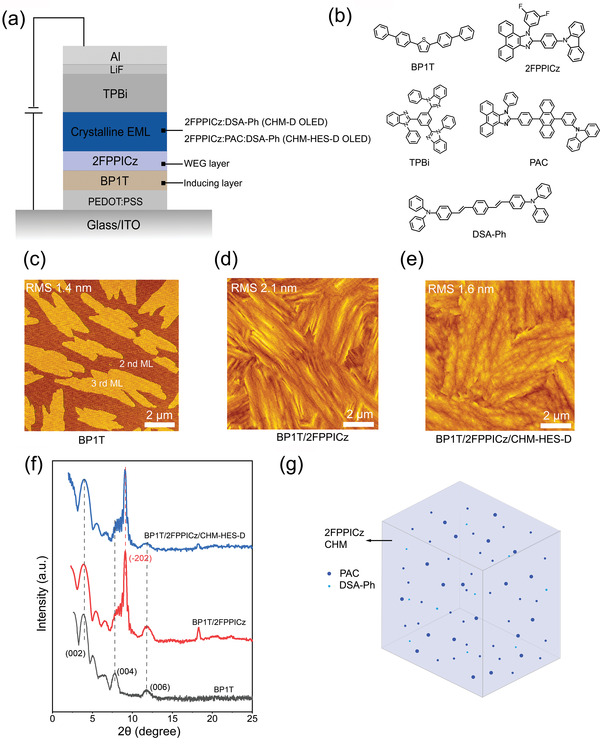
Structure of the OLEDs and characterization of crystalline thin films. a) Schematic configuration of the devices. b) Molecular formulas of the materials used in the devices. c) AFM image of BP1T crystalline thin film. d) AFM image of 2FPPICz crystalline host matrix. e) AFM image of 2FPPICz CHM‐HES‐D thin film. f) Out‐of‐plane XRD patterns of BP1T crystalline thin film, 2FPPICz crystalline film, and 2FPPICz CHM‐HES‐D film. g) Schematic illustration of the 2FPPICz CHM‐HES‐D thin film.

The application of 2‐(4‐(9H‐carbazol‐9‐yl)phenyl)‐1‐(3,5‐difluorophenyl)‐1H‐phenanthro[9,10‐d]imidazole (2FPPICz) wide‐gap fluorescent material^[^ ^15^, ^38^ ^]^ and the “hot exciton” material 2‐(4‐(10‐(3‐(9H‐carbazol‐9‐yl)phenyl) anthracen‐9‐yl)phenyl)‐1‐phenyl‐1H‐phenanthro[9,10‐d] imidazole (PAC)^[^ ^29^ ^]^ have been studied in the previous reports. In this paper, 2FPPICz was selected as the crystalline host matrix (CHM), the “hot exciton” material PAC as a sensitizer (HES), and a fluorescent dopant (D) 1‐4‐di‐[4‐(*N*,*N*‐diphenyl)amino]styryl‐benzene (DSA‐Ph) as an emitter with PLQY 0.91. Two types of doped devices (CHM‐D OLED, and CHM‐HES‐D OLED) are fabricated to explore the impact of “hot exciton” material as a sensitizer and its effect on harvesting triplet excitons in C‐OLED. In the CHM‐D device, only the dopant DSA‐Ph was doped in the CHM to form the emitting layer (EML). In the EML of CHM‐HES‐D, PAC sensitizer and DSA‐Ph were co‐doped in the CHM. The structure of devices and molecular formulas used in the devices are illustrated in **Figure** **1**a,b. The energy‐level diagram of the materials is shown in Figure S1 (Supporting Information), respectively. These two doped C‐OLEDs have similar structures and preparation processes. First, a 40 nm thick PEDOT: PSS layer on ITO is used as the anode and smooth substrate for the growth of the crystalline thin film. A 6 nm thick 2,5‐di([1,1“‐biphenyl]‐4‐yl)thiophene (BP1T) crystalline thin film is selected as the holes transport layer (HTL) and electrons block layer (EBL) due to its hole‐transport ability and suitable energy levels in OLEDs.^[^ ^14^, ^15^ ^]^ A 5 nm thick 2FPPICz crystalline thin film is also used as the holes transport layer and simultaneously acts as the growing base of 2FPPICz CHM. The 2FPPICz and the 2 wt% DSA‐Ph are co‐deposited on the 2FPPICz crystalline substrate to form a 20 nm thick EML of the CHM‐D device. In the EML preparation process of the CHM‐HES‐D device, 15 wt% PAC and 2 wt% DSA‐Ph are co‐deposited with 2FPPICz CHM. All crystalline thin films are prepared at a substrate temperature of 102 °C. Then, a 30 nm thick amorphous 2,2”,2“”‐(1,3,5‐benzinetriyl)‐tris(1‐phenyl‐1‐H‐benzimidazole) (TPBi), 1 nm thick LiF, and 150 nm thick Al, were fabricated to act as the electron transport layer, electron injection layer, and cathode, respectively.

It has been confirmed that the weak epitaxy growth (WEG) of 2FPPICz crystalline thin films can be applied on surfaces of smooth amorphous substrates, and their crystal structures are consistent for either PEDOT: PSS or Si/SiO_2_ substrate.^[^ ^15^, ^39^, ^40^ ^]^ Thus, the crystalline thin films were prepared on Si/SiO_2_ substrate by WEG method,^[^ ^41^, ^42^ ^]^ to facilitate the analysis and investigation on the crystalline EML morphologies. More than two monolayers BP1T (less than three layers) were first deposited on the Si/SiO_2_ substrate to form a continuous crystalline thin film at substrate temperature 102 °C, acting as an inducing layer for the subsequent epitaxy growth of crystalline thin films. Atomic force microscopy (AFM) images demonstrate that the root‐mean‐square (RMS) surface roughness of the BP1T layer is only 1.4 nm, providing a smooth crystalline substrate for the growth of the WEG layer (Figure 1c). The 5 nm thick 2FPPICz crystalline WEG layer deposited on the BPIT inducing layer held at 102 °C, still maintained continuity and molecular smoothness (RMS 2.1 nm) (Figure 1d) and served as the subsequent growth of 2FPPICz CHM. Afterward, a 20 nm thick double‐doped system crystalline EML in which “hot exciton” PAC sensitizer (15 wt%), and blue dopant DSA‐Ph (2 wt%) are co‐doped in 2FPPICz CHM at the same substrate temperature 102 °C. That is, three materials, 2FPPICz, PAC, and DSA‐Ph were evaporated simultaneously from three independent evaporation sources and deposited on top of the 5 nm thick 2FPPICz WEG thin film, forming the CHM‐HES‐D structure. The CHM‐HES‐D thin film shows a high degree of order with molecular level roughness (RMS 1.6 nm) (Figure 1e). Out‐of‐plane X‐ray diffraction (XRD) is used to determine the crystal structural characteristics of these crystalline films. The XRD patterns of the crystalline thin films, including BP1T (6 nm), BP1T (6 nm)/2FPPICz (25 nm), and BP1T (6 nm)/2FPPICz (5 nm)/CHM‐HES‐D (20 nm) are shown in Figure 1f. The 25 nm thick 2FPPICz thin film is composed of well‐connected domains with oriented stripe‐like crystals, similar crystal morphology to the 5 nm thick 2FPPICz crystalline thin film, as shown in Figure S2a (Supporting Information). The doped crystalline thin films remain well‐connected crystal domains and have the RMS roughness of about 2 nm until the PAC doping concentration reaches 15 wt% (Figure S2b–d, Supporting Information). In addition, using a flexible polyethylene terephthalate (PET)/ITO/PEDOT: PSS as a flexible substrate, 2FPPICz crystalline films were grown on top to characterize the adaptability to the flexible substrate, as shown in Figure S3a (Supporting Information). Compared to the initial status (Figure S3d, Supporting Information), the morphology of 2FPPICz crystalline thin film after bending 100 times with the radius of 1 cm has not changed (Figure S3e, Supporting Information), indicating that the crystalline thin film is able to afford substrate deformation which is similar to amorphous thin films. The detailed description of the flexibility test is in Note S1 (Supporting Information). The BP1T and 2FPPICz crystal structures have been studied in the previous reports.^[^ ^14^, ^15^, ^40^ ^]^ (002), (004), and (006) planes of BP1T crystalline thin film and (‐202) plane of 2FPPICz crystalline thin film have been marked at their corresponding peaks, respectively. The XRD patterns of BP1T/2FPPICz/CHM‐HES‐D are consistent with BP1T/2FPPICz and no new diffraction peak appears. The results reveal that PAC sensitizer and blue dopant distributed in the 2FPPICz crystalline host matrix are amorphous and without destroying the crystal structure of the 2FPPICz crystalline matrix. The schematic illustration of CHM‐HES‐D thin film composed of the crystalline host matrix, “hot exciton” PAC sensitizer, and dopant molecules is shown in Figure 1g.

**Figure 2:**
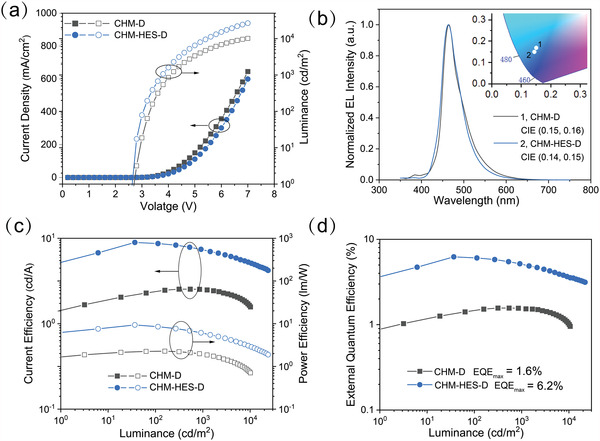
EL performance of CHM‐D and CHM‐HES‐D OLEDs. a) Current density (left)–luminance (right)–voltage curves. b) EL spectra and the corresponding CIE of the devices at 1000 cd m^−2^. c) Luminance‐dependent CE characteristics (left) and Luminance‐dependent PE characteristics (right). d) Luminance‐dependent EQE curves of the devices.

**Figure** **2**a shows the voltage‐dependent current density and luminance curves of the devices. It can be clearly observed that the CHM‐HES‐D OLED has a higher luminance than that of the CHM‐D OLED at the same driving voltage, the brightness of the CHM‐HES‐D OLED can be up to 1000 cd m^−2^ at a voltage of 3.8 V. The CHM‐D and CHM‐HES‐D devices achieve pure blue emission with peaks of 463 nm, with a similar full width at half maximum (FWHM) of ≈50 nm. Their corresponding CIE is (0.15, 0.16) and (0.14, 0.15), respectively (Figure 2b). Figure 2c demonstrates the current efficiency (CE) and power efficiency (PE) of the devices, achieving a maximum PE of 2.3 and 9.4 lm W^−1^, respectively. The maximum EQE of the CHM‐D OLED is only 1.6%. Through introducing the PAC sensitizer, the maximum EQE was enhanced to 6.2% in the CHM‐HES‐D device (Figure 2d). The EQE of OLEDs can be described as follows

(1) $$\mathrm{EQE}=\gamma \times \eta_{\gamma }\times \Phi_{\mathrm{PLQY}}\times \eta_{\mathrm{out}}$$

where *γ* is the charge recombination factor, *η_*γ*_* is the radiative exciton production efficiency (25% for conventional fluorescent materials), *η*_out_ is the light‐out‐coupling efficiency, and Ф_PLQY_ is the PLQY of the emitter molecule (0.91 for DSA‐Ph). Assuming a general light output coupling of 20% and a charge recombination factor of ideally 100%, the maximum EQE of the only fluorescent dye DSA‐Ph emitter is limited to 4.6%. The CHM‐HES‐D OLED has evidently broken through this upper limit, suggesting that a large number of triplet excitons have been converted to singlets in the EL process of 2FPPICz CHM‐HES‐D OLED. The key performance of these two devices is summarized in **Table** **1**.

**Table 1:** Key performance parameters of CHM‐D OLED and CHM‐HES‐D OLED

Devices	*V*_on_/*V*_1000_/∆*V* ^a)^ [V]	CE_max_/PE_max_/EQE_max_ ^b)^ [cd A^−1^/lm W^−1^/%]	CE/PE/EQE^c)^ [cd A^−1^/lm W^−1^/%]	*λ*_max_ ^d)^ [nm]	FWHM [nm]	CIE [X, Y]
CHM‐D OLED	2.7/4.1/1.4	2.6/2.3/1.6	2.5/1.9/1.5	463	50	(0.15, 0.16)
CHM‐HES‐D OLED	2.6/3.8/1.2	8.9/9.4/6.2	7.2/5.9/5.0	463	50	(0.14, 0.15)

^a)^ V_on_ and V_1000_ are operation voltage at a luminance of 1 and 1000 cd m^−2^, respectively; ∆*V* is the difference between *V*_on_ and *V*_1000_;

^b)^ Maximum CE/PE/EQE values;

^c)^ CE/PE/EQE values at a brightness of 1000 cd m^−2^;

^d)^ Maximum emission wavelength.

**Figure 3:**
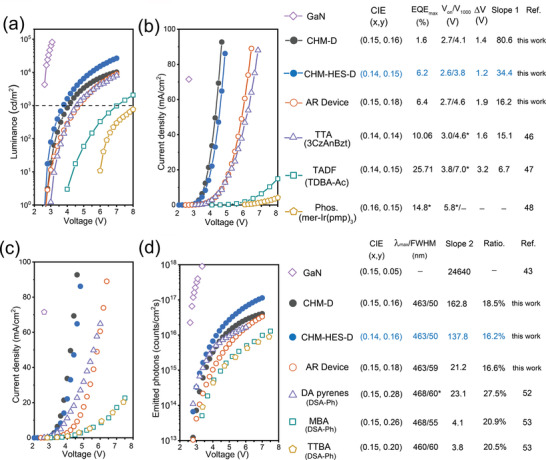
Comparisons of CHM‐HES‐D and CHM‐D with amorphous thin‐film OLEDs. a) Comparison of Luminance versus Voltage curves. b) Voltage (*V*)‐dependent current density of OLEDs. ∆*V* = *V*_1000_(1000 cd m^−2^) − *V*_0_(1 cd m^−2^). c) Comparison of Current density versus voltage curves. d) Comparison of voltage‐dependent semi‐log emitted photons (*N*). All comparative data are extracted from their corresponding references. * Estimated values based on figures in the corresponding literature.

For inorganic blue LEDs such as InGaN/GaN devices,^[^ ^43^ ^]^ their high carrier mobility and exciton utilization efficiency result in high photons output. Considering similar principles in organic semiconductors, 2FPPICz crystalline thin film possesses a high hole carrier mobility of ≈ 0.10 cm^2^V^−1^s^−1^ and electron carrier mobility of ≈ 0.015 cm^2^V^−1^s^−1^,^[^ ^15^ ^]^ much larger than amorphous layers (10^−8^ to 10^−2^ cm^2^ V^−1^ s^−1^) in the present amorphous thin‐film OLEDs (A‐OLEDs).^[^ ^44^, ^45^ ^]^ The comparison of the brightness of the CHM‐HES‐D and CHM‐D OLEDs (2 wt% DSA‐ph) with reported typical blue A‐OLEDs (CIE_y_ ≤ 0.15) with high EQE, including, TTA,^[^ ^46^ ^]^ TADF,^[^ ^47^ ^]^ and phosphorescent^[^ ^48^ ^]^ are shown in **Figure** **3**a. The CHM‐HES‐D OLED shows the lowest turn‐on voltage and the fastest brightness climb as the driving voltage increases, suggesting that the CHM‐HES‐D device has the smallest operation voltage at the same brightness after the devices are turned on. The brightness of the CHM‐HES‐D device switches from 1 to 1000 cd m^−2^ with only an increment of 1.2 V, revealing the advantageous capability in quickly reaching lighting status. The CHM‐HES‐D and CHM‐D OLEDs also show much faster ramping of current density as the driving voltage is increased (Figure 3b). Slope 1 is the instantaneous slope of current density (*J*)*–*driving voltage (*V*) curves at their corresponding luminance of about 1000 cd m^−2^, that is, the areal differential conductance of these OLEDs. The 2FPPICz CHM‐HES‐D OLED has much higher areal differential conductance, indicating that the high mobility of the crystalline thin film is beneficial to improving the conductance of the OLEDs and achieving a fast turning‐on at low operation voltage. In contrast with all amorphous thin‐film OLEDs with similar color purity, the CHM‐HES‐D OLED has a lower ratio of series resistance Joule heat to input power of only 10.7% at 1000 cd m^−2^, that is, producing the minimal heat loss ratio at the operation state. A comparison of several electric parameters is summarized in Table S1 (Supporting Information).

In addition, an amorphous reference device ITO/PEDOT: PSS/BP1T (6 nm)/2FPPICz (5 nm)/2FPPICz: PAC (15 wt%): DSA‐Ph (2 wt%)/TPBi (30 nm)/LiF/Al (marked as AR device) was fabricated. As shown in Figure S4a (Supporting Information), compared to the AR device (turn‐on voltage of 2.7 V and an increment voltage (∆*V*) of 1.9 V from 1 to 1000 cd m^−2^), the CHM‐HES‐D device shows a lower turn‐on voltage (2.6 V), the faster brightness climb as the driving voltage increases (∆*V* of 1.2 V). The CHM‐HES‐D device also shows a much faster ramping of current density as the driving voltage is increased. Compared with the EL spectrum of the AR device, the EL spectrum of the CHM‐HES‐D device is narrowed by about 10 nm, showing higher color purity (Figure S4b, Supporting Information). Benefiting from the capability of “hot exciton” material PAC harvesting both singlet/triplet excitons, the AR device can achieve the maximum EQE of 6.4% similar to that of the CHM‐HES‐D device (Figure S4d, Supporting Information). Morphological stability of thin films is a key factor of influencing devices’ operational stability,^[^ ^8^ ^]^ especially in the high‐temperature conditions in which the device operates.^[^ ^49^, ^50^, ^51^ ^]^ Therefore, we studied the stability of the 2FPPICz: PAC (15 wt%): DSA‐Ph (2 wt%) crystalline thin film (marked as CHM‐HES‐D thin film) and the 2FPPICz: PAC (15 wt%): DSA‐Ph (2 wt%) amorphous reference thin film (marked as AR thin film) at **Condition 1** (25 °C; humidity, 55%RH–65%RH; without encapsulation) and **Condition 2** (a temperature of 80 °C and pressure of 300 Pa; without encapsulation), respectively. The morphology of the CHM‐HES‐D thin film has nearly no changes or phase separation when the CHM‐HES‐D thin film was placed for 300 h at **Condition 1** and for 200 h at **Condition 2** (Figure S5a–k, Supporting Information). Figure S5l (Supporting Information) shows the out‐of‐plane XRD patterns of CHM‐HES‐D thin film after being maintained for 200 h, 300 h at **Condition 1**, and 100 h, 200 h at **Condition 2**, showing the diffraction patterns of the thin film and thin film structures have not changed compared to the initial status. On the other hand, the AR thin film shows that the phenomenon of phase separation and molecules aggregation appears in the time of only several hours at **Condition 1** and **Condition 2**, respectively, and the phase separation gets more serious as time increases (Figure S6, Supporting Information). These results reveal that compared to the amorphous thin film, the CHM‐HES‐D thin film possesses good thermal stability, which is potentially beneficial to constructing long‐lifetime OLED devices. The detailed description of morphology stability is in Note S2 (Supporting Information).

The several A‐OLEDs with DSA‐Ph emitters doped in different hosts (CIE*_y_* < 0.3),^[^ ^52^, ^53^ ^]^ are contrasted with CHM‐HES‐D OLED (Figure 3c,d). The CHM‐HES‐D device exhibits a narrower FWHM and higher color purity. The amount of emitted photons per unit time per unit area (*N*) is adopted to objectively evaluate the photon output ability of these OLEDs. N is calculated from the following equation

(2) N=EQE*J/e

in which *e* represents the elementary charge, and *J* is the current density. It can be easy to understand *N* can be used as an evaluation standard to eliminate the impact of human visual function.^[^ ^15^ ^]^ Figure 3c first shows a voltage‐dependent current density of these OLEDs. Benefiting from the low series resistance, CHM‐D and CHM‐HES‐D devices have larger current density characteristics when driving voltage was applied, indicating that the holes and electrons are easily injected and transmitted under the same electric field. According to the computational formula of *N*, the large photon output is determined by the high EQE and high conductance of OLEDs. Figure 3d shows a voltage‐dependent semi‐log *N* of these OLEDs. Because of the high carrier mobility of crystalline thin films and the collection excitons capacity of “hot excitons” material PAC, CHM‐HES‐D OLED has prominent conductance and sufficient EQE. CHM‐HES‐D device achieves the largest amount of photons emitted at the same operation voltage compared to the reported high‐efficiency DSA‐Ph amorphous thin‐film OLEDs and is closest to the photoemission output capability of inorganic InGaN/GaN LED^[^ ^43^ ^]^ among these OLEDs. Slope 2 and Ratio. represent the instantaneous slope of *J–V* curves and the series resistance Joule heat to input power of these devices at a driving voltage of 5 V, respectively. A comparison of these electric parameters is summarized in Table S1 (Supporting Information). The description of detailed calculation process on Joule heat to input power is Note S3 (Supporting Information). The CHE‐HES‐D device possesses the predominant conductance and the lower Joule heat loss (16.2%) in various DSA‐Ph amorphous devices. These results demonstrate that by combining crystalline thin‐film host with “hot exciton” sensitizers, the C‐OLED can produce an efficient blue emission and a much stronger and faster photon output than various high‐efficiency A‐OLEDs.

**Figure 4:**
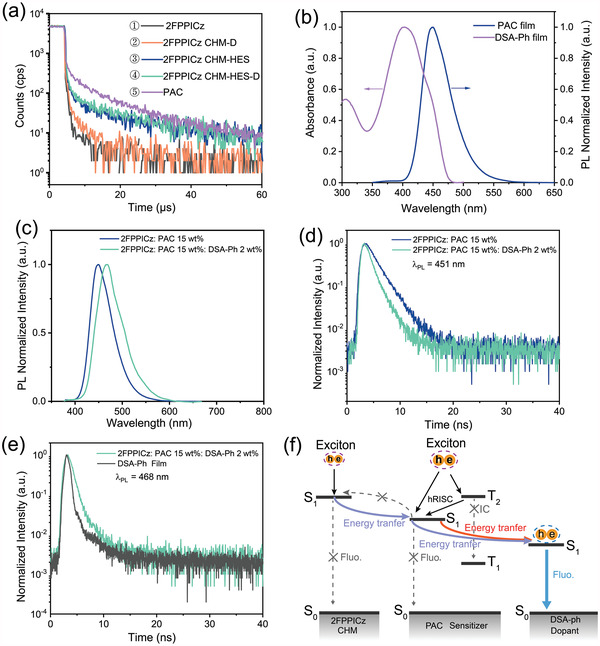
EL transient decay curves, absorption and PL spectra, PL transient decay curves, and schematic diagram of energy transfer. a) EL transient decay curves of CHM‐D, CHM‐HES‐D, and several reference OLEDs were measured at a driving voltage of 5 V. b) Absorption of DSA‐Ph film (left) and PL spectra of PAC film prepared at substrate temperature 102 °C. c) PL spectra of CHM‐HES (15 wt%) and CHM‐HES (15 wt%)‐D (2 wt%) films. d) Transient PL decay curves of CHM‐HES (15 wt%) and CHM‐HES (15 wt%)‐D (2 wt%) at an emission of 451 nm. e) Transient PL decay curves of the DSA‐Ph film and CHM‐HES (15 wt%)‐D (2 wt%) at an emission of 468 nm. f) Schematic diagram of the PAC sensitization processes in CHM‐HES‐D OLED.

To understand the sensitizing process of “hot exciton” PAC in CHM‐HES‐D OLED, several similar reference OLEDs including 2FPPICz C‐OLED (marked as Device ①, emitter: 2FPPICz), CHM‐HES (15 wt%) OLED (marked as Device ③, emitter: PAC) and PAC A‐OLED (marked as Device ⑤, emitter: PAC), were prepared to perform the transient EL analysis. The device structures and EL spectra of these reference OLEDs are shown in Figure S7 (Supporting Information). The transient EL characteristic curves of these reference OLEDs, the CHM‐D (2 wt%) device (marked as Device ②, emitter: DSA‐Ph), and CHM‐HES (15 wt%)‐D (2 wt%) (marked as Device ④, emitter: DSA‐Ph) are shown in **Figure** **4**a. For Device ① and Device ②’ EML only consisting of conventional fluorescent materials, their transient EL decay curves show fast decay characteristics. When the “hot exciton” material PAC of 15 wt% was introduced in 2FPPICz CHM (Device ③ and Device ④), their transient EL spectra exhibit similar behavior to that of Device ⑤ using only PAC in EML, a longer decay compared to the cases of EMLs without any PAC materials. These results indicate that triplet/singlet excitons in 15 wt% PAC sensitizer can be finally harnessed for radiative transition (CHM‐HES device) or be transferred to DSA‐Ph for radiative transition (CHM‐HES‐D device). On the other hand, compared to the transient EL decay curve of Device ⑤ (100% PAC in EML), the ratio of the prompt and delayed EL part of Device ③ (15 wt% PAC in EML) increases and decreases, respectively (Figure 4a). This result shows that compared to all excitons formed on PAC molecules in Device ⑤, part excitons can still be formed on 2FPPICz molecules when the holes and electrons were injected into EML and then the singlet excitons of 2FPPICz were transferred to the S_1_ state of the PAC molecules in Device ③. To further analyze the formation process of excitons in Device ③, several CHM‐HES devices with different PAC doping concentrations (2FPPICz: PAC *x* wt %, *x* = 0, 5, 10, 15) were fabricated (Figure S8a, Supporting Information). The EL spectra of CHM‐HES devices (10 and 15 wt%) exhibit the same spectra as Device ⑤ (emitter: PAC) and only the EL spectrum of the low doping concentration (5 wt%) CHM‐HES device has a slight 2FPPICz emission band at less than 400 nm (Figure S8b, Supporting Information). However, as the PAC doping concentration changes from 5 wt% to 15 wt%, the maximum EQE of CHM‐HES OLEDs is gradually increased (Figure S8c, Supporting Information), implying that more excitons are formed in PAC sensitizer as PAC doping concentration increases. Figure S8d (Supporting Information) shows the schematic diagram of energy transfer between 2FPPICz and PAC in the CHM‐HES device (15 wt%). In addition, to confirm the process of the energy transfer process between 2FPPICz and PAC in CHM‐HES OLED, various crystalline thin films of 2FPPICz: PAC (*x* wt%, *x* = 5, 10, 15) (marked as CHM‐HES films) were prepared. The absorption spectra of PAC film, PL spectra of 2FPPICz crystalline thin film, PAC pure film, and several CHM‐HES films are shown in Figure S9a,b (Supporting Information). The PL spectrum of 2FPPICz crystalline film overlaps the absorption of PAC film, implying the S_1_ state exciton energy of 2FPPICz molecules can be resonantly transferred to the S_1_ states of PAC molecules through the energy transfer process. The PL spectrum of 5 wt% PAC doped CHM‐HES film with 2FPPICz emission band is similar to the EL spectrum of 5 wt% PAC doped CHM‐HES device. The PL spectra of CHM‐HES films with the PAC concentration from 10 to 15 wt% are the same as the PL spectrum of PAC pure film, and no emission band of 2FPPICz crystalline appears, suggesting the existence of a sufficient and efficient energy transfer process from 2FPPICz CHM to PAC molecules. PL transient decay curves measurements also proved this process of efficient energy transfer. As shown in Figure S9c (Supporting Information), by monitoring the emission decay of 2FPPICz crystalline film and CHM‐HES films at 404 nm (The emission peak of 2FPPICz), the lifetime decay curve of CHM‐HES film is lower than that of 2FPPICz crystalline film. The ratio of the delayed part of the CHM‐HES film gradually increases as the concentration of PAC is increased from 5 wt% to 10 wt% through monitoring the emission peak at 451 nm (The emission peak of PAC) (Figure S9d, Supporting Information). The slightly decreased lifetime of PAC emission in the doping concentration of 15 wt% CHM‐HES film should be attributed to the concentration quenching effects of the fluorescent dyes.^[^ ^20^ ^]^ These results show the efficient energy transfer process between 2FPPICz and PAC in CHM‐HES OLED.

When DSA‐Ph of 2 wt% was introduced into CHM‐HES (15 wt%) films, several CHM‐HES (15 wt%)‐D (2 wt%) thin films were prepared to investigate the energy transfer process between CHM‐HES and DSA‐Ph. Figure 4b exhibits the UV−vis absorption spectrum of PAC and PL spectrum of DSA‐Ph, the partial overlap of the two spectra indicating that the energy transfer can be well expected. Compared to the emission peak of CHM‐HES (15 wt%) crystalline film at 451 nm, the CHM‐HES (15 wt%)‐D (2 wt%) shows emission peaks of 468 nm (Figure 4c), showing that an efficient energy transfer occurs from 2FPPICz CHM‐HES to DSA‐Ph. Figure 4d,e shows the transient PL of these thin films, by monitoring the emission peak at 451 nm (Dominant emission peak of CHM‐HES film) and 468 nm (Dominant emission peak of DSA‐Ph), respectively. The ratio of the delayed part of the CHM‐HES‐D films curves gradually decreases at 451 nm and increases at 468 nm, respectively, verifying the efficient energy transfer process from CHM‐HES to blue emitter DSA‐Ph. Hereto, a schematic diagram of the PAC sensitization processes and energy transfer process in the CHM‐HES‐D system under electrical excitation can be described (Figure 4f). Singlet excitons from 2FPPICz (first transferred to PAC through the energy transfer process) and triplet/singlet excitons harvested by PAC sensitizer are transferred to the high PLQY blue dopant DSA‐Ph via energy transfer to achieve an efficient blue emission.

## 3 Conclusions

In summary, we have developed a new general route to improve the performance of conventional fluorescent blue‐emission crystalline thin‐film OLED (C‐OLEDs) by doping “hot exciton” PAC sensitizers to harvest both singlet/triplet excitons. When the “hot exciton” sensitizer (HES) and high‐PLQY emitting dopant (D) are co‐doped into the crystalline host, the continuity of the crystalline host matrix (CHM) can be well maintained. The CHM‐HES‐D OLED in this work achieved an efficient emission with a maximum EQE of 6.2%. In the meantime, owing to the high transport properties of crystalline thin films, CHM‐HES‐D OLED can achieve a faster turn‐on, a lower ratio of series resistance Joule heat to input power, and a higher photon output capacity compared to reported typical high‐EQE amorphous thin‐film OLEDs (A‐OLEDs) with blue emission (CIEy ≤ 0.15). This work demonstrates that the strategy of crystalline thin films combined with the “hot exciton” sensitizing dopants will provide a promising way for developing the next‐generation OLEDs with high performance.

## 4 Experimental Section

### Materials

BP1T, 2FPPICz, and “Hot exciton” materials PAC were synthesized according to the previous reports and their energy levels can be found in the previous works.^[^ ^14^, ^29^, ^38^, ^54^ ^]^ The energy levels of DSA‐ph and TPBi were from ref. [55] and ref. [56], respectively, both were purchased from Luminescence Technology Corp. (Lumtec). All materials were purified twice by thermal gradient sublimation before using.

### Film and Device Fabrication

The preparation process of crystalline thin films and devices that adopted similar methods were introduced in the previous reports.^[^ ^8^, ^14^, ^15^ ^]^ To investigate the morphologies and crystal structures of crystalline thin‐film and doped crystalline thin film, the heavily doped n‐type silicon wafers with a 300 nm thermally oxidized SiO_2_ layer (capacitance per unit area (*C_i_*) = 10 nF cm^−2^) were used as the substrates. Acetone, alcohol, and deionized water were used to clean the Si/SiO_2_ substrates in turn, and then desiccated in high purity nitrogen and dried in a bake oven at 120 °C for 20 min. A 120 nm thick NPB amorphous thin film with 2 wt% DSA‐Ph was prepared on quartz substrates, which were cleaned using the same method as Si/SiO_2_ substrates, for measuring the photoluminescence quantum yield (PLQY) of DSA‐Ph. The crystalline OLED devices were fabricated on the 180 nm thick anodes composed of a metal oxide based on indium tin oxide (ITO) with 10 Ω per square. Before depositing of organic materials in the vacuum chamber, ITO substrates were first purged with detergent for 30 min, and then ultrasonicated in acetone, alcohol, and deionized water in turn for 30 min. After being dried in a bake oven at 120 °C, ITO substrates were treated with oxygen plasma for 15 min. Then, PEDOT: PSS (Clevious P Al 4083) was spin‐coated to modify the anode at 4000 rpm for 30 s and then dried in a bake oven at 120 °C for 30 min. Finally, the Si/SiO_2_, quartz, or ITO was transferred into a vacuum chamber at a pressure of under 10^−4^ Pa. The growth rates of the BP1T crystalline inducing layer and 2FFPICz crystalline epitaxy thin film were ≈4–10 Å min^−1^ at a substrate temperature of 102 °C. The growth rates of NPB amorphous thin film, electron transfer layer TPBi, LiF, and Al were 1–2, 1–2, 0.05–0.08, and 10–15 Å S^−1^, respectively, at the room temperature. The thicknesses of all organic thin films were monitored by a quartz‐crystal microbalance. The effective emission area of each device that was overlapping the area between the ITO and Al electrodes was 4.0 mm × 4.0 mm.

### Film and Device Characterization

Using an SPI 3800/SPA 300 HV (Seiko Instruments Inc., Japan) atomic force microscope (AFM) to investigate the morphologies of the thin films in tapping mode. By employing a thin‐film diffraction diffractometer (D8 Discover) with Cu K*α* radiation (*λ* = 1.54056 Å) at 40 kV and 40 mA (Bruker, Germany), the out‐of‐plane X‐ray diffraction (XRD) patterns were acquired. To measure the photoluminescence (PL) spectra and transient PL decay curves, Edinburgh Instrument FLS980 spectrometer was used. Using a Shimadzu UV‐3600 spectrometer, UV–vis absorption spectra were acquired. The PLQY property of DSA‐Ph was measured by using a Hamamatsu Photonics C9920‐2. By using a Keithley source measurement system (Keithley 2400/2000) with a calibrated silicon photodiode, the *J*–*V*–*L* characteristics of OLED devices were tested under an ambient atmosphere. The electroluminescence (EL) spectra were recorded with a Spectra scan PR650 spectrophotometer. In addition, based on the current density, luminance, and electroluminescence spectra, the external quantum efficiency (EQE) values were acquired assuming a Lambertian distribution. All thin films and device characterizations were performed under an ambient laboratory atmosphere at room temperature.

## Conflict of Interest

The authors declare no conflict of interest.

## Author Contributions

F.Z. and D.H.Y. initiated and designed the research. J.J.Y. carried out the growth of the crystalline thin films and the fabrication and characterization of the OLEDs. W.T.Z. contributed to the characterization of morphologies of crystalline thin films. D.H.H. and Y.G.M. contributed to the synthesis of materials. J.J.Y., F.Z., and D.H.Y. prepared the manuscript with input from all authors. F.Z. and D.H.Y. supervised the project. All authors discussed the results and commented on the manuscript.

## Supporting information

Supporting Information

## Data Availability

The data that support the findings of this study are available from the corresponding author upon reasonable request.
