# Supplementary material for: An Efficient Blue‐Emission Crystalline Thin‐Film OLED Sensitized by “Hot Exciton” Fluorescent Dopant
Source: Adv Sci (Weinh). 2022 Nov 17;10(2):2203997. doi: 10.1002/advs.202203997 (PMC9839864; doi:10.1002/advs.202203997)
Supplement: Supplementary file 1 — Supporting Information [file ADVS-10-2203997-s001.pdf]

ADVANCED SCIENCE
Open Access

Supporting Information

for *Adv. Sci.*, DOI 10.1002/advs.202203997

An Efficient Blue-Emission Crystalline Thin-Film OLED Sensitized by "Hot Exciton" Fluorescent Dopant

Jingjie Yang, Wantao Zheng, Dehua Hu, Feng Zhu, Yuguang Ma and Donghang Yan*

Supporting Information

An Efficient Blue-Emission Crystalline Thin-Film OLED Sensitized by "Hot Exciton" Fluorescent Dopant

Jingjie Yang[1,2], *Wantao Zheng*[1,2], *Dehua Hu*[3], *Feng Zhu*[1,2*], *Yuguang Ma*[3], *Donghang Yan*[1,2]

[1]State Key Laboratory of Polymer Physics and Chemistry, Changchun Institute of Applied Chemistry, Chinese Academy of Sciences, Changchun 130022, China.

[2]School of Applied Chemistry and Engineering, University of Science and Technology of China, Hefei 230026, China.

[3]State Key Laboratory of Luminescent Materials and Devices, South China University of Technology, Guangzhou 510640, China.

*Correspondence and requests for materials should be addressed to F.Z. (Email: zhufeng@ciac.ac.cn)

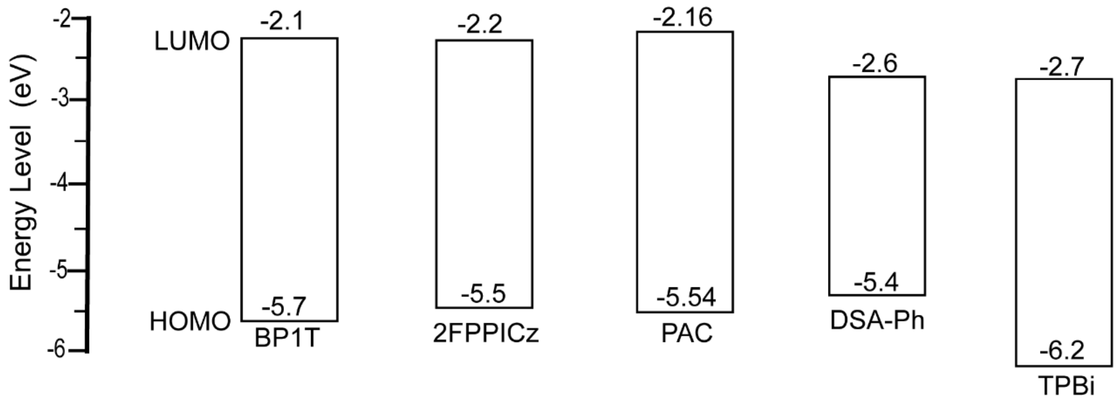

Fig. S1. Energy levels of materials used in the devices.

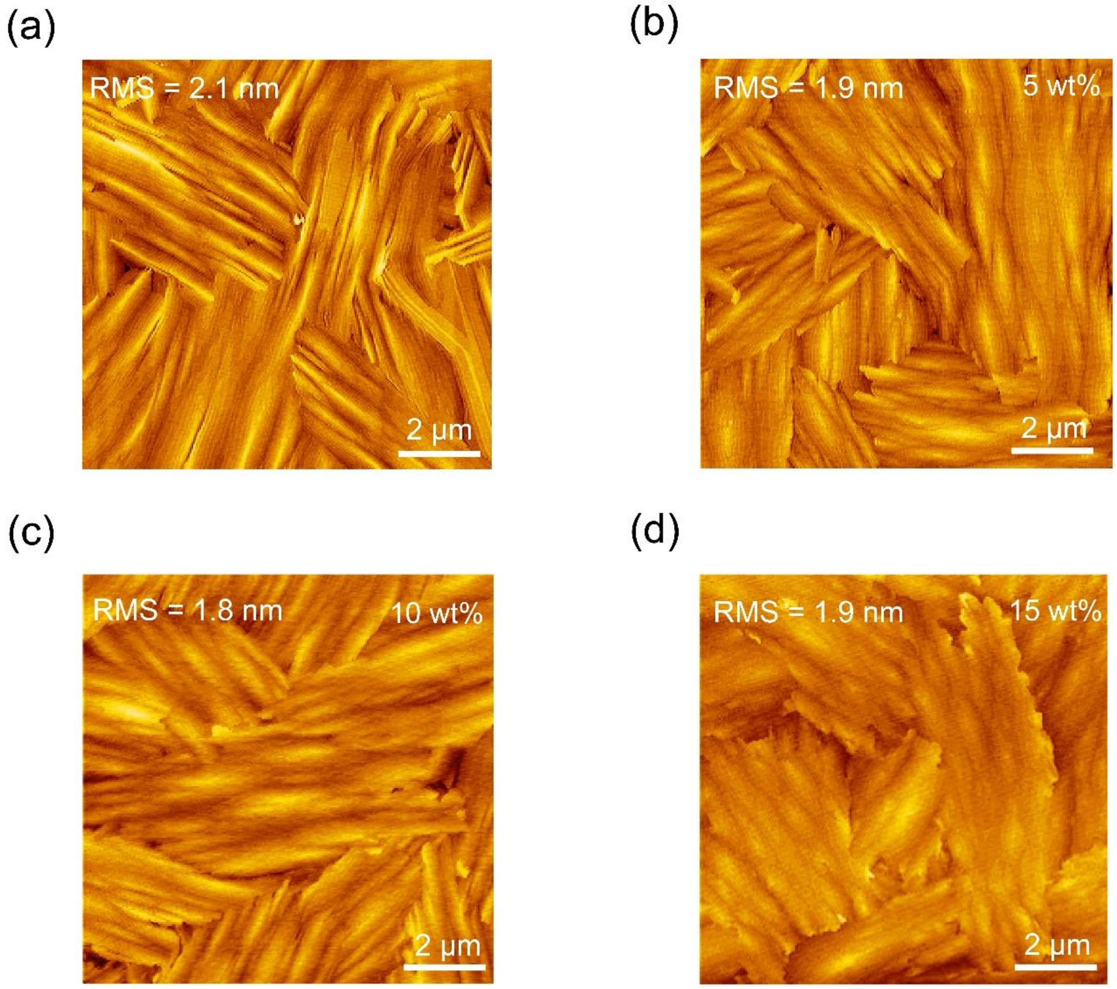

Fig. S2. AFM images of crystalline thin films. (a) AFM image of 25-nm thick 2FPPICz crystalline thin film. (b-d) AFM images of 2FPPICz crystalline thin film: PAC x wt%, x = 5 (b), x = 10 (c), x = 15 (d).

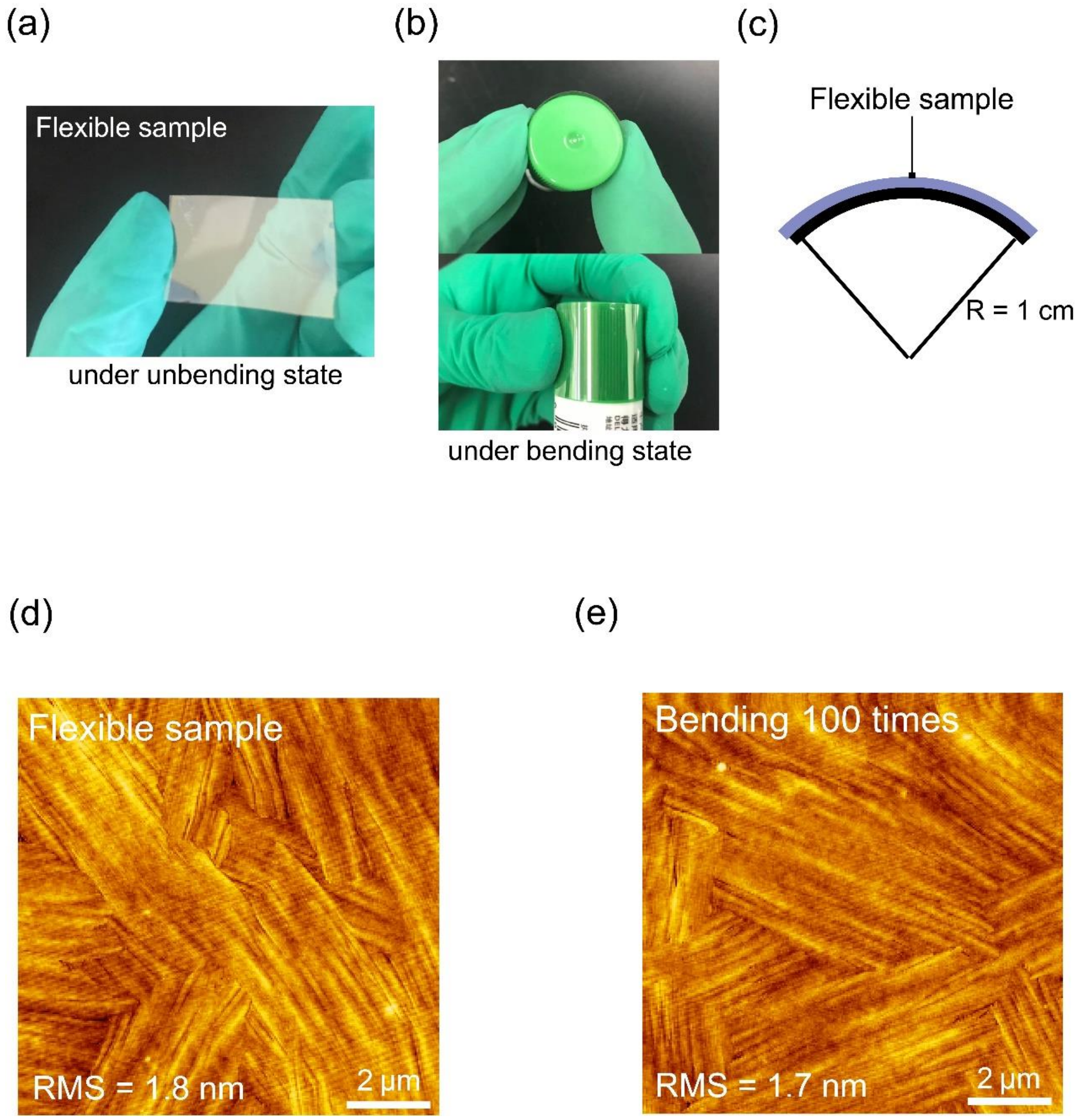

Fig. S3. Flexibility test of 25-nm thick 2FPPICz crystalline thin film. (a) Flexible sample under an unbending state. (b) Flexible sample under bending state with a bending radius of 1 cm. (c) Bending schematic diagram of 2FPPICz crystalline thin film. (d) AFM image of 2FPPICz crystalline thin film before bending. (e) AFM image of 2FPPICz crystalline thin film after bending for 100 times.

Note S1. Description of flexibility test of the 2FPPICz crystalline thin film.

Polyethylene terephthalate (PET) (0.175 mm)/ITO (185 nm) was chosen as a flexible substrate, then an approximately 40-nm thick PEDOT: PSS layer was spin-coated on the surface for carrying out the subsequent growth of crystalline thin films. The 6-nm thick BP1T first was deposited on PET/ITO/PEFOT: PSS at 102 °C substrate temperature, then 25-nm thick 2FPPICz was deposited on BP1T crystalline thin film hold 102 °C. As shown in Fig. S3a, a photograph of the 25-nm 2FPPICz crystalline thin film on a flexible substrate is presented under an unbending state. Fig. S3d shows the AFM image of 2FPPICz crystalline thin film on the flexible substrate before bending, and it can be observed that the 2FPPICz thin film is composed of well-connected domains with oriented stripe-like crystals. Then, taking a cylinder with a radius of 1 cm as the bending standard, the 2FPPICz crystalline thin film on the flexible substrate was bent for 100 times (Fig. S3b). The schematic diagram is shown in Fig. S3c, and R represents the bending radius of the crystalline thin film. Then AFM measurement was carried out to characterize the thin film after bending. As shown in Fig. S3e, compared to the initial morphology of the 2FPPICz crystalline thin film, the morphology of 2FPPICz crystalline thin film after bending 100 times has not changed, indicating that the crystalline thin film is able to afford substrate deformation which is similar to amorphous thin films.

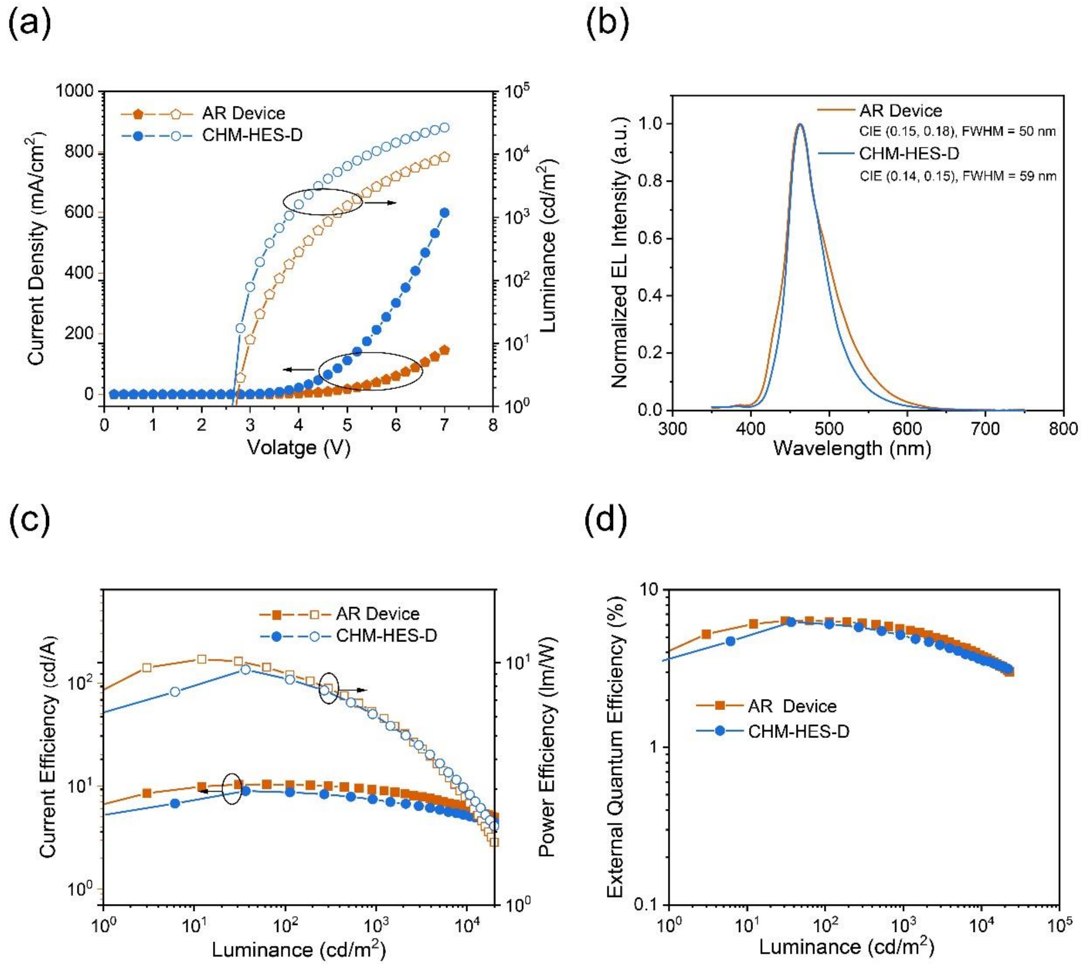

Fig. S4. EL performance of CHM-HES-D and amorphous device with the emitter of 2FPPICz: PAC (15 wt%): DSA-Ph (2 wt%) (Marked as AR Device). (a) Current density (left) - Luminance (right) - Voltage curves. (b) EL spectra and corresponding CIE of the devices at 1000 cd/m^2. (c) Luminance-dependent CE characteristics (left) and Luminance-dependent PE characteristics (right). (d) Luminance-dependent EQE curves of the devices.

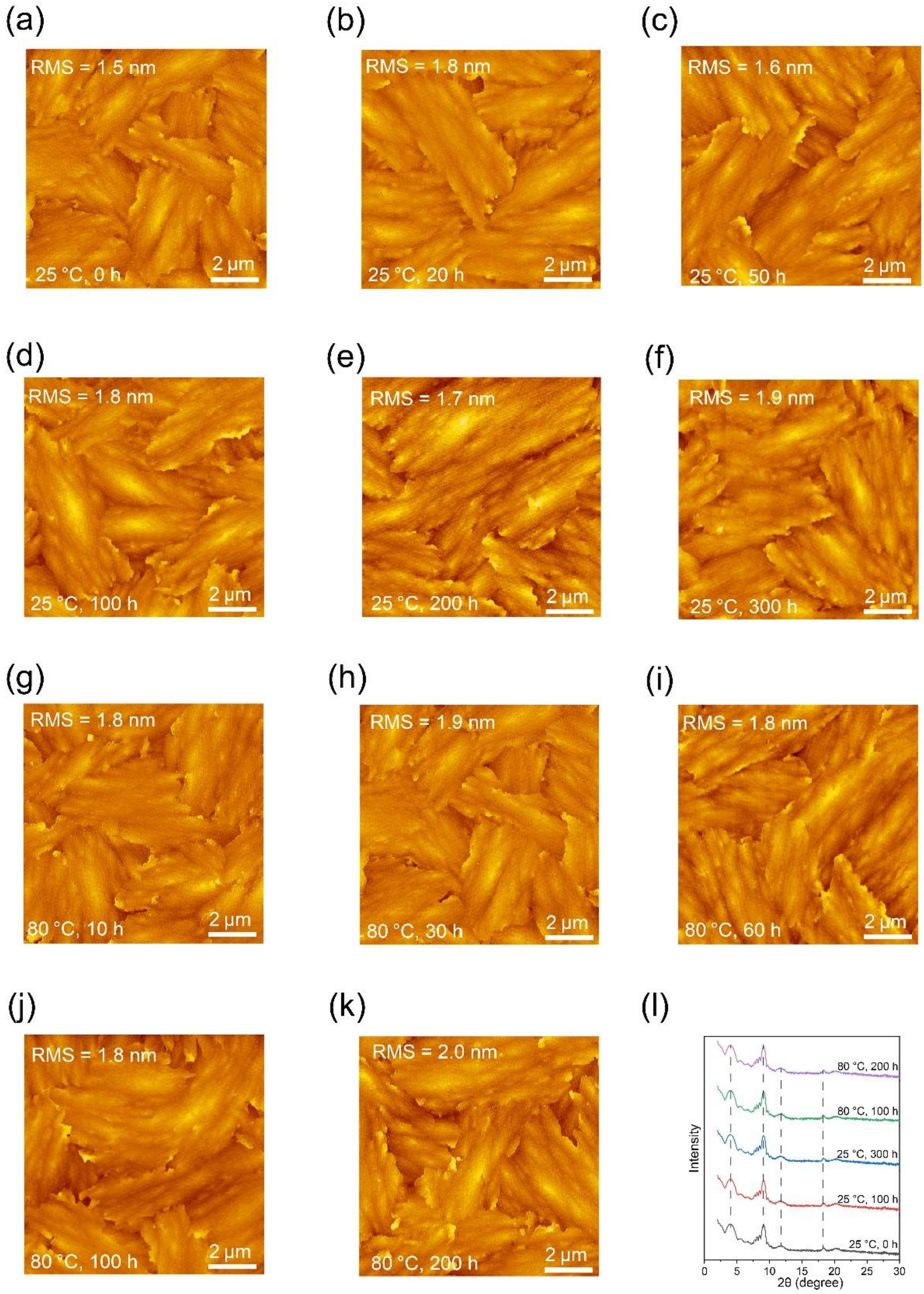

Fig. S5. Morphology evolution of CHM-HES-D thin film. (a-f) Time-dependent AFM images of CHM-HES-D thin film with the time of 0 h (a), 20 h (b), 50 h (c), 100 h (d), 200 h (e), 300 h (f) at Condition 1. (g-k) Time-dependent AFM images of CHM-HES-D thin film with the time of 10 h (g), 30 h (h), 60 h (i), 100 (j), and 200 h (k) at Condition 2. (l) Out-of-plane XRD patterns of CHM-HES-D thin film.

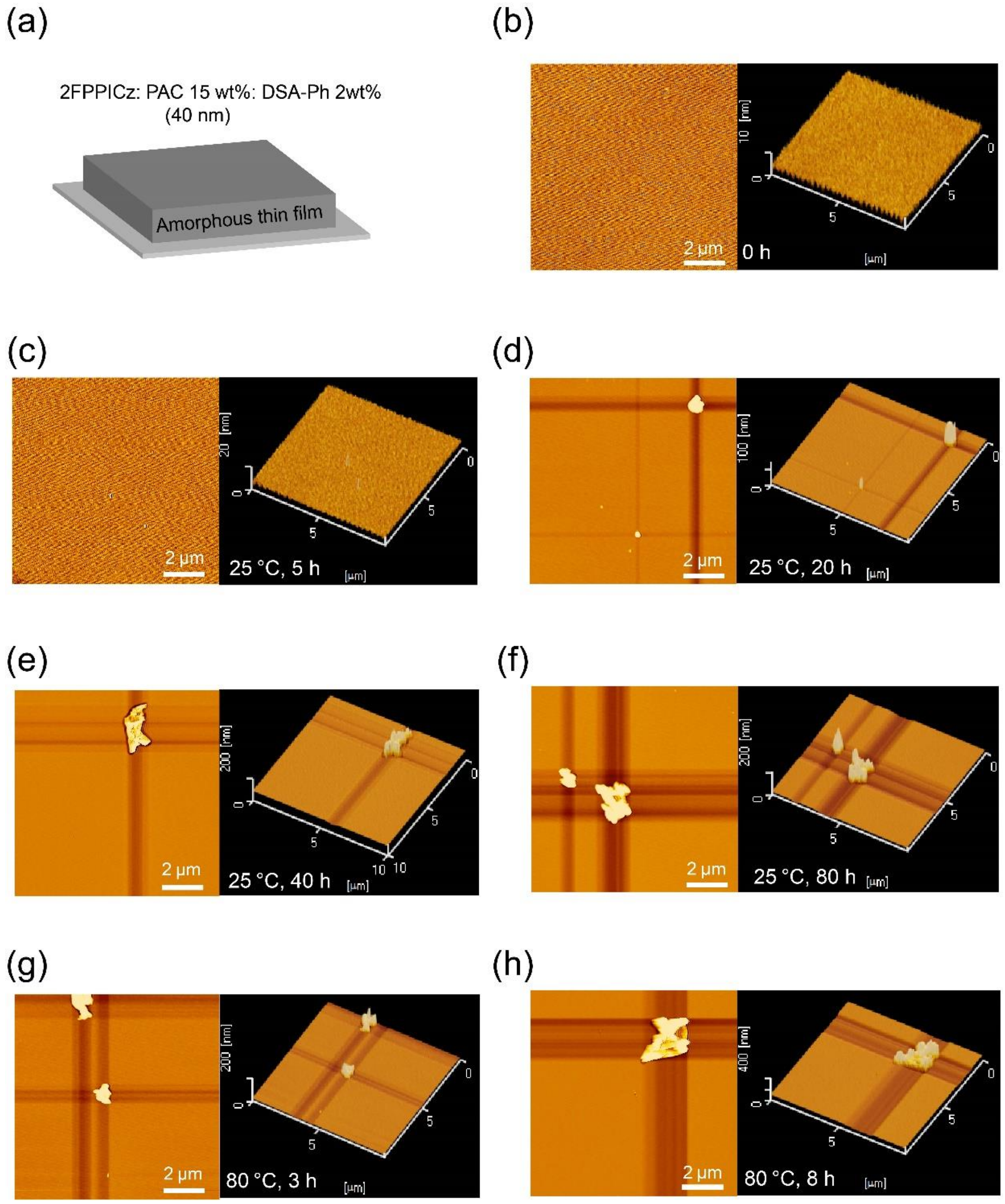

Fig. S6. Morphology evolution of AR thin film. (a) Structure of the amorphous reference 2FPPICz: PAC (15 wt%): DSA-Ph (2 wt%) (40 nm) (AR) thin film. (b-f) Time-dependent AFM images of the AR thin film with the time of 0 h (b), 5h (c), 20 h (d), 40 h (e), and 80 h (f) at 25 °C. (g-h) Time-dependent AFM images of the AR thin film with the time of 3 h (g), 8 h (h) at 80 °C.

Note S2. Description of morphology stability of the CHN-HES-D thin film and AR

thin film

The 25-nm thick 2FPPICz: PAC (15 wt%): DSA-Ph (2 wt%) (mark as CHM-HES-D thin film) on Si/SiO_2 / BP1T crystalline thin film (6nm) / 2FPPICz crystalline substrate (10 nm). Firstly, the time-dependent morphology evolution of CHM-HES-D thin film by AFM characterization in an atmospheric environment (**Condition 1:** 25 °C; humidity, 55 %RH~65 %RH; without encapsulation). Fig. S5a-f shows the morphology evolution of CHM-HES-D thin film with the time of 0 h (a), 20 h (b), 50 h (c), 100 h (d), 200 h (e), and 300 h (f). At the beginning of T = 0 h, the CHM-HES-D thin film with a small RMS roughness of about 2 nm was demonstrated. As the time increases to 300 h (f), no obvious changes are happening in thin-film morphology, and RMS is maintained at approximately 2 nm. In addition, it is observed that no phase separation occurs, showing the CHM-HES-D thin film has great morphology stability. As shown in Fig. S5g-k, the AFM images of CHM-HES-D thin film were placed in an oven (**Condition 2:** at a temperature of 80°C and pressure of 300 Pa), with the time of 10 h (g), 30 h (h), 60 h (i), 100 (j), 200 h (k). It can be observed that the morphology of the CHM-HES-D thin film has not changed compared to the initial status. Fig. S5l shows the out-of-plane XRD patterns of CHM-HES-D thin film after being maintained for 200 h, 300h at **Condition 1**, and 100 h, 200 h at **Condition 2**, and the patterns of the thin film have not changed compared to the initial status. To further reveal the advantage of crystalline thin-film morphology, a reference amorphous thin film 2FPPICz: PAC (20 wt%): DSA-Ph (2 wt%) (40 nm) was prepared, which is named AR thin film. Fig. S6 shows the morphology evolution of the AR thin film **at Condition 1** with the time of 0 h (b), 5h (c), 20 h (d), 40 h (e), and 80 h (f). The flat and uniform amorphous thin film can be observed from the AFM images at the initial status. However, the phenomenon of phase separation and molecule aggregation appears in the time of only several hours. The phase separation gets more serious as time increases and aggregates are about several hundred nanometers when the thin film was placed for 80 hours. The morphology evolution of the AR thin film at **Condition 2** was also studied, of which

the significant phase separation occurs just within several hours (in Fig. S6g, h). In summary, these results reveal that compared to the amorphous thin film, the CHM-HES-D thin film possesses good thermal stability, which is beneficial to realizing long-lifetime OLED devices.

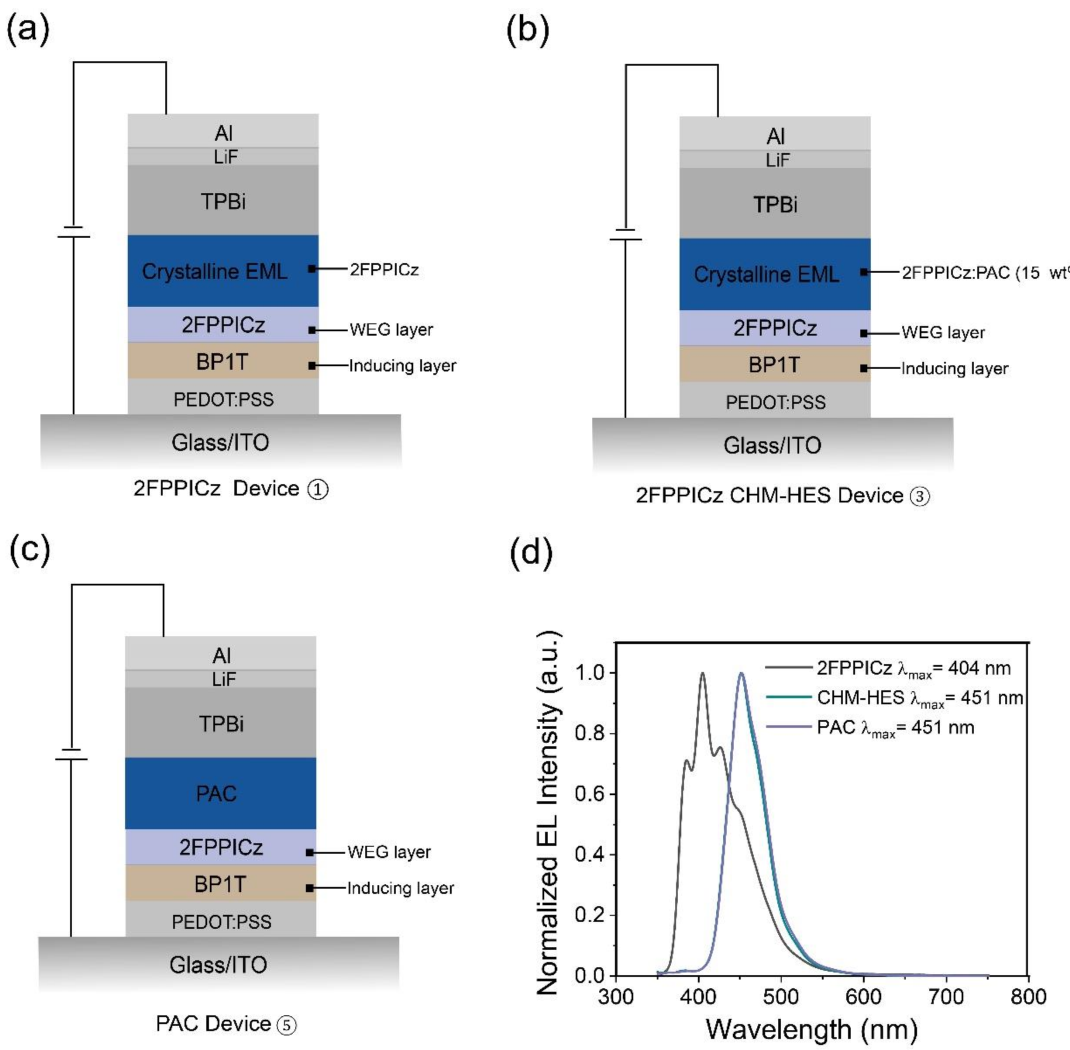

Fig. S7. Structures and EL spectra of devices. (a) Schematic diagram of CHM-HES OLED structure. (b) Schematic diagram of the 2FPPICz OLED structure. (c) Schematic diagram of PAC OLED structure. (d) EL spectra of the devices at 5 V.

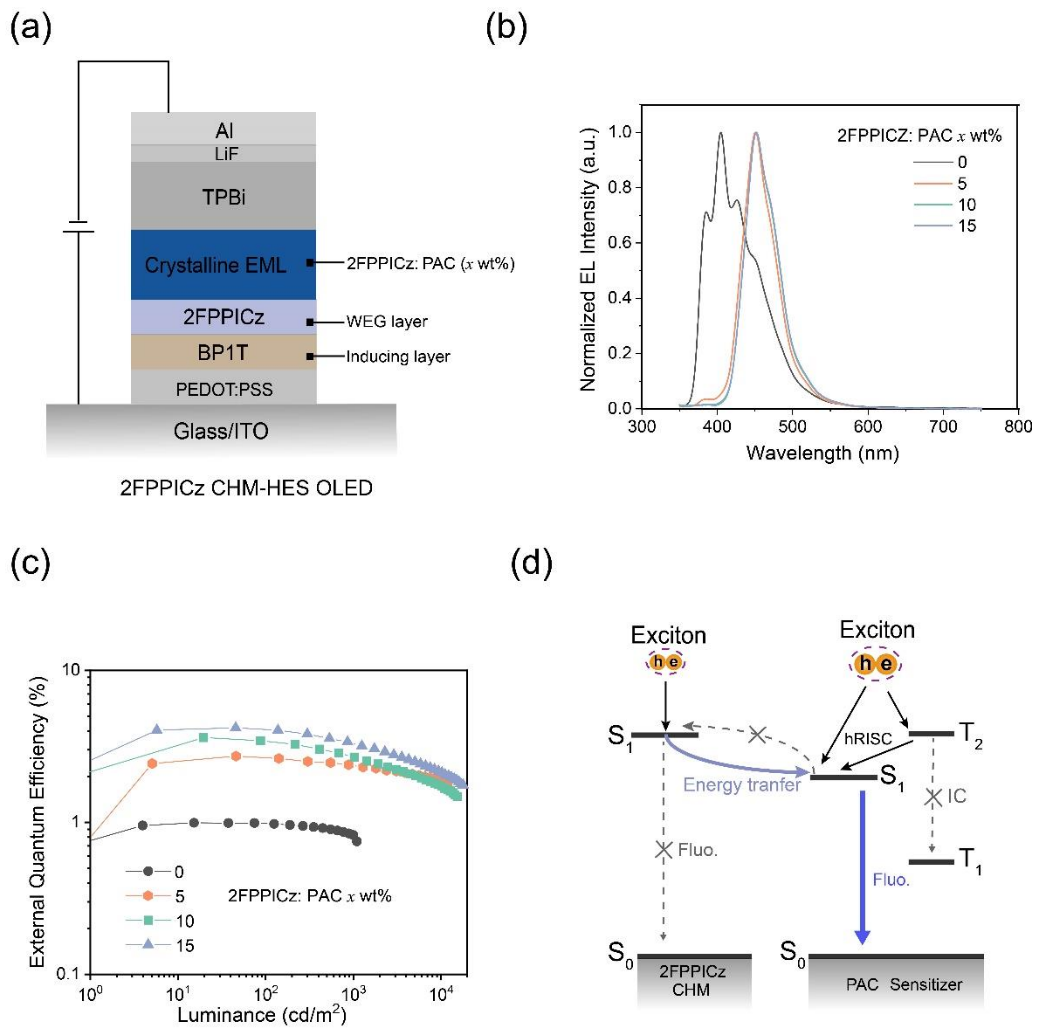

Fig. S8. Structure and performance of the devices. (a) Schematic diagram of the device structure. (b) EL spectrum of the devices at 5 V. (c) EQE-Luminance curves of the devices. (d) Schematic diagram between 2FPPICz and PAC.

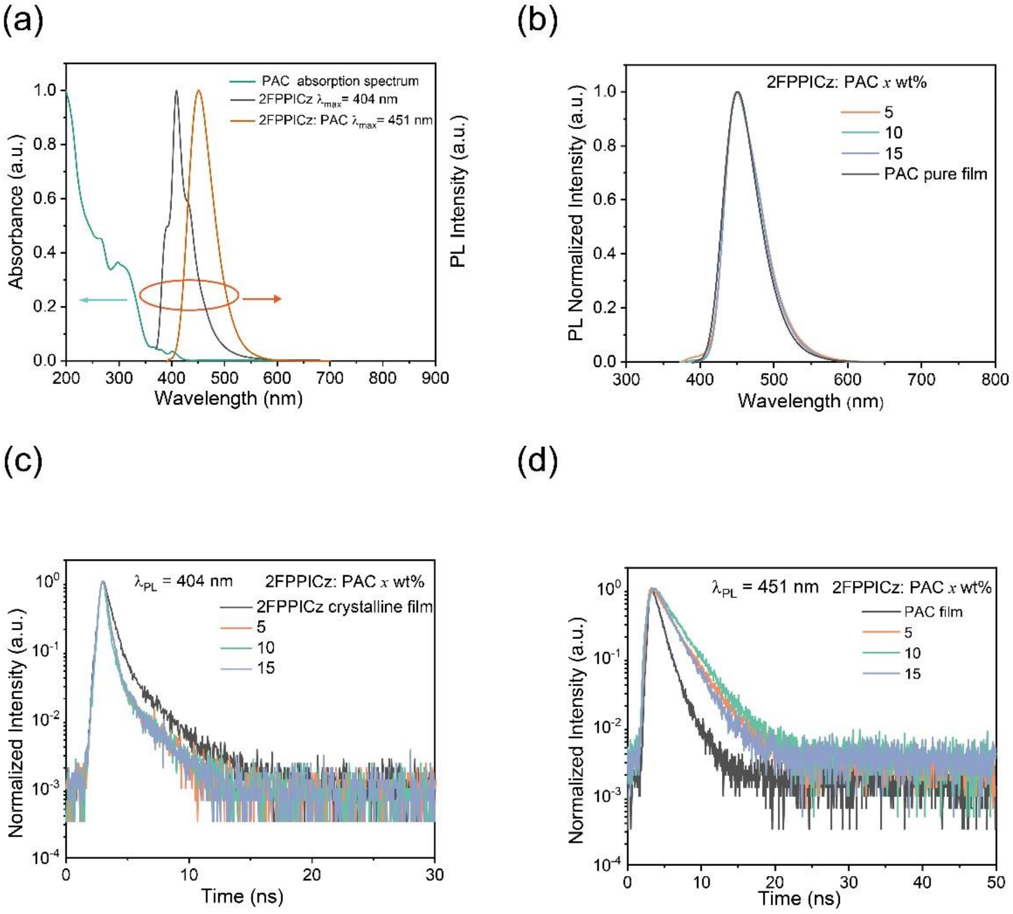

Fig. S9. Absorption and PL spectra, and PL transient decay curves. (a) Absorption of PAC film (left), PL spectrum of 2FPPICz, and PL spectrum of PAC film. Note: 2FPPICz crystalline thin film was prepared on BP1T crystalline layer on quartz substrate at 102 °C. PAC film was prepared on quartz substrate at 102 °C. The excitation wavelength of the PL spectra is 350 nm. (b) PL spectra of CHM-HES films consisting of 2FPPICz: PAC x wt% (x = 5, 10, 15), and PL spectrum of PAC film prepared at substrate temperature 102 °C. (c) Transient PL decay curves of 2FPPICz crystalline film and CHM-HES films consisting of 2FPPICz: PAC x wt% (x = 5, 10, 15) at an emission peak of 404 nm. (d) Transient PL decay curves of the PAC film and CHM-HES films consisting of 2FPPICz: PAC x wt% (x = 5, 10, 15) at an emission peak of 451 nm.

Table S1. | Summary of areal Joule heat loss of the CHM-D and CHM-HES-D OLED and other typical amorphous thin film blue-emission OLEDs at approximately 1000 cd/m^2

Emitters	Voltage (V)	Current density (mA/cm^2)	Input power (mW/cm^2)	Differential conductance (mS/cm^2)	Differential resistance (kΩ cm^2)	Joule heat loss (mW/cm^2)	Joule heat loss ratio (%)	CIE (x,y)
CHM--D (DSA-Ph) This work	4.1	38.8	159.1	80.6	0.0124	18.7	11.7	(0.15, 0.16)
CHM-HES-D (DSA-Ph) This work	3.8	14.0	53.2	34.4	0.0291	5.7	10.7	(0.14, 0.15)
AR Device(DSA-Ph) This work	4.6	9.3	42.8	16.2	0.0617	5.4	12.6	(0.15, 0.18)
TTA (3CzAnBzt) Ref. 46	4.6	8.6	39.6	15.1	0.0662	4.9	12.4	(0.14, 0.14)
TADF (TDBA-Ac) Ref. 47	7.0	6.4	44.8	6.7	0.149	6.1	13.6	(0.14, 0.15)
**Summary of areal Joule heat loss of the CHM-D and CHM-HES-D OLED and other A-OLEDs with DSA-Ph emitters doped in different hosts (CIEy< 0.3) at a driving voltage of 5 V.**								
CHM--D (DSA-Ph) This work	5	150.5	752..5	162.8	0.00614	139.1	18.5	(0.15, 0.16)
CHM-HES-D (DSA-Ph) This work	5	111.8	559.0	137.8	0.00726	90.7	16.2	(0.14, 0.15)
AR Device Device (DSA-Ph) This work	5	17.6	88.0	21.2	0.0472	14.6	16.6	(0.15, 0.18)
DA pyrenes (DSA-Ph) Ref. 52	5	31.8	159.0	23.1	0.0433	43.8	27.5	(0.15, 0.28)
MBA	5	4.3	21.5	4.1	0.243	4.5	20.9	(0.15,

(DSA-Ph)<br>Ref. 53								0.26)
TTBA<br>(DSA-Ph)<br>Ref. 53	5	3.9	19.5	3.8	0.263	4.0	20.5	(0.15, 0.20)

Note S3. Detailed calculation on ratios of Joule-heat loss to input power

The actual current-voltage curve of OLED can be described by the Shockley equation:

$$I - \frac{(V - IR_S)}{R_P} = I_S\, exp^{\frac{e(V-IR_S)}{n_{ideal}kT}} \quad \text{(S1)}$$

where R_s is the series resistance, R_p is the parallel resistance, I_s is the reverse saturation current, n_{ideal} is the ideality factor of the diode, k is the Boltzmann constant, and T is the thermodynamic temperature. Series resistance (R_s) of the OLED can be described by the slope of the current-voltage curve under high driving voltage:

$$R_S = \frac{dV}{dI} \quad \text{(S2)}$$

When current flows through the OLED device, the internal resistance characteristics of the device generates Joule heat. Therefore, the parallel resistance (R_p) can be ignored when the OLED is operating at a large driving voltage. The only series resistance (R_s) of the OLED is considered when the Joule heat of the OLED can be calculated as below equation:

$$P_J = I^2 R_S \quad \text{(S3)}$$

The input power is the product of current and voltage:

$$P_{input} = IV \quad \text{(S4)}$$

In the end, the "Ratio" can be calculated with the following equation:

$$Ratio = \frac{P_J}{P_{input}} \quad \text{(S5)}$$
